# Development and validation of a keypoint region-based convolutional neural network to automate thoracic Cobb angle measurements using whole-spine standing radiographs

**DOI:** 10.1007/s00701-025-06645-x

**Published:** 2025-08-23

**Authors:** Mert Marcel Dagli, Jonathan H. Sussman, Jaskeerat Gujral, Bhargavi R. Budihal, Marie Kerr, Jang W. Yoon, Ali K. Ozturk, Patrick J. Cahill, Jason Anari, Beth A. Winkelstein, William C. Welch

**Affiliations:** 1 Department of Neurosurgery, Perelman School of Medicine, University of Pennsylvania, Philadelphia, PA USA; 2 Department of General Medicine, BGS Global Institute of Medical Sciences, Bengaluru, India; 3 Department of Orthopaedic Surgery, Perelman School of Medicine, University of Pennsylvania, Philadelphia, PA USA; 4 Department of Bioengineering, University of Pennsylvania, Philadelphia, PA USA

**Keywords:** Humans, Spinal curvatures, Adolescent, Artificial intelligence, Spinal fusion, Neural

## Abstract

**Purpose:**

Adolescent idiopathic scoliosis (AIS) affects a significant portion of the adolescent population, leading to severe spinal deformities if untreated. Diagnosis, surgical planning, and assessment of outcomes are determined primarily by the Cobb angle on anteroposterior spinal radiographs. Screening for scoliosis enables early interventions and improved outcomes. However, screenings are often conducted through school entities where a trained radiologist may not be available to accurately interpret the imaging results.

**Methods:**

In this study, we developed an artificial intelligence tool utilizing a keypoint region-based convolutional neural network (KR-CNN) for automated thoracic Cobb angle (TCA) measurement. The KR-CNN was trained on 609 whole-spine radiographs of AIS patients and validated using our institutional AIS registry, which included 83 patients who underwent posterior spinal fusion with both preoperative and postoperative anteroposterior X-ray images.

**Results:**

The KR-CNN model demonstrated superior performance metrics, including a mean absolute error (MAE) of 2.22, mean squared error (MSE) of 9.1, symmetric mean absolute percentage error (SMAPE) of 4.29, and intraclass correlation coefficient (ICC) of 0.98, outperforming existing methods.

**Conclusion:**

This method will enable fast and accurate screening for AIS and assessment of postoperative outcomes and provides a development framework for further automation and validation of spinopelvic measurements.

## Introduction

Adolescent idiopathic scoliosis (AIS) affects 2–4% of the adolescent population, causing a three-dimensional deformity of the spinal column that can impair normal motion and posture, potentially leading to lung and heart dysfunction, early-onset osteoarthritis, and disc degeneration if untreated [34, 36]. Spinal fusion surgery in progressive cases remains the main treatment option, but variations in pre-operative characteristics, surgical implants, and maneuvers result in a wide range of outcomes, with approximately 20% being less than satisfactory [7, 35].

Accurate assessment of spinopelvic parameters, particularly the Cobb angle, is essential for optimizing surgical strategies and enhancing patient outcomes [10, 14, 26, 27]. Part of the planning process includes reviewing whole-spine standing radiographs to measure these parameters [9, 13, 23]. However, traditional TCA measurements using coronal radiographs are error-prone due to subjective interpretation, variability among practitioners, and differences in training, necessitating improved methodologies [10, 26, 27]. This variability can lead to imprecise evaluations, highlighting the need for more reliable methods. Moreover, screenings are often conducted at schools, where there is often limited access to radiologists with training in interpreting the relevant images. This could lead to an increased rate of false-positive and false-negative screening tests that could hinder patient outcomes or yield increased healthcare costs [8, 31]. In this study, we investigated the development and validation of a keypoint region-based convolutional neural network (KR-CNN) for the automated measurement of the coronal TCA. KR-CNNs facilitate image analysis by identifying specific points on an image through a multi-step classification task. KR-CNN operate through a multi-step process. Initially, the network applies a series of convolutional layers to extract high-level features from the input image. This is followed by the generation of a region proposal network, in which potential regions of interest are identified where keypoints may be located. These keypoints are specific, identifiable points or features within an image, in this case representing anatomic landmarks. These regions are then refined and classified by the network to precisely define structures. This method enables the accurate identification of recurring patterns and shapes in the setting of variable backgrounds. Consequently, this is particularly well-suited for radiographic measurement tasks in which differences in imaging settings can obscure the identification of well-defined structures. Calculating the TCA angle involves identifying the bony structures of the spine, followed by the extension of lines within a plane. These measurements assist in surgical decision-making and treatment planning for patients with AIS, thereby improving surgical outcomes and patient satisfaction. Overall, the goal of this National Institutes of Health R21-funded feasibility study was to demonstrate the utility of the KR-CNN approach for the fast, accurate, and generalizable measurement of spinopelvic features.

## Methods

### Guidelines

The methodology and reporting of our study were supported by established guidelines, including the Strengthening the Reporting of Observational Studies in Epidemiology (STROBE), the Transparent Reporting of a Multivariable Prediction Models for Individual Prognosis or Diagnosis + Artificial Intelligence (TRIPOD + AI), and the Checklist for Artificial Intelligence in Medical Imaging (CLAIM) [4, 6, 25].

### Data source

The Penn Medicine and Children's Hospital of Philadelphia (CHOP) Institutional Review Board (IRBs 852054, 22–020186) granted approval and authorized the review of Electronic Health Records (EHR). All methods were performed in accordance with relevant guidelines and regulations. Due to the retrospective design of the study, the need for direct patient consent was waived. The database, “Dataset 16,” which was used to train the KR-CNN to automate coronal TCA measurements, was requested from SpineWeb and consisted of 609 anterior–posterior spinal X-ray images annotated with keypoints for Cobb angle determination as part of the Accurate Automated Spinal Curvature Estimation (AASCE) 2019 Grand Challenge [5, 37]. For the validation, an institutional registry for AIS correction surgery was constructed at our center, involving manual chart reviews (MCR) and the integration of medical imaging data from our Picture Archiving and Communication System (PACS). The creation of this registry was a collective endeavor under the multi-institutional umbrella of the Penn Medicine Healthcare System. Prior to the transfer of data from CHOP, all necessary data sharing agreements were established and finalized.

### Patient selection and outcomes

Database records of surgeries performed between June 13, 2014, and December 19, 2022, were reviewed, to include pediatric patients (age < 18 years old) who underwent elective thoracolumbosacral posterior spinal fusion (PSF) for AIS correction. Exclusion criteria were age at surgery above 18 years, inclusion of cervical regions in the fusion construct, or surgical intervention performed for any indication other than deformity, such as tumor, trauma, or infection. The primary outcome of this study was the development and validation of a framework to fully automate coronal TCA measurements. Secondary outcomes included comparison of model performance to other models prior reported in the literature.

### Missing data

The prevalence of missingness in tabular data was 0%, and a complete-case approach was used. No imputations were performed. All records included in model development and validation had complete imaging data.

### Statistics, model development, and external validation

**Fig. 1:**
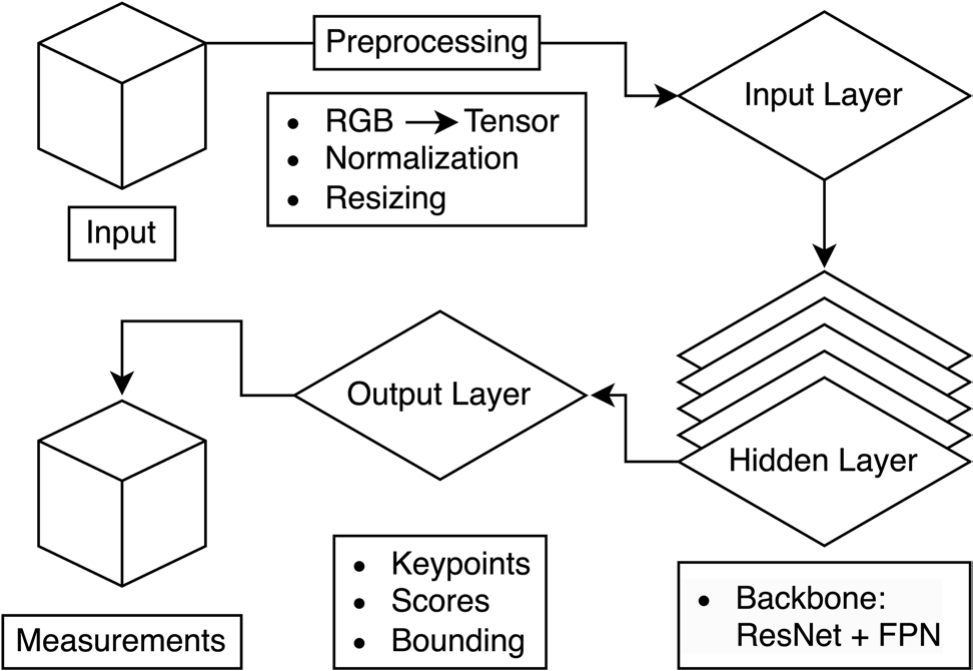
Overview of the developed and validated keypoint region-based convolutional neural network (KR-CNN) architecture

Baseline characteristics were summarized using descriptive statistics. Dataset 16 from SpineWeb was used to train the model. For preprocessing, bounding boxes were derived from keypoint locations, ensuring compatibility with the KR-CNN model. Images were normalized, resized, and transformed into tensors. Low-confidence detections were filtered using confidence thresholding and non-max suppression to retain only relevant instances. The hidden layers used a ResNet + FPN as their backbone. The output layer included information on keypoints, confidence scores, and bounding boxes. Using derived information from the output layer, a function was utilized to automate the calculation of radiographic measurements using extracted coordinates (Fig. 1). Validation was performed using our institutional registry. Both radiographic automated measurements and manual measurements were utilized to calculate performance metrics of the measurement automation tool. These metrics included mean absolute error (MAE), median absolute error (MedAE), mean squared error (MSE), symmetric mean absolute percentage error (SMAPE), and the intraclass correlation coefficient (ICC). Percentile-based bootstrapping with 10,000 resamples was used to calculate 95% confidence intervals for the MedAE. All development, statistical analysis, and the creation of figures were performed using Python, version 3.11 (Python Foundation, Wilmington, DE).

## Results

### Primary outcomes

The KR-CNN with a ResNet-50-FPN backbone was trained using “Dataset 16,” which consists of 609 spinal anterior–posterior (coronal) X-ray images with annotated keypoints needed to calculate the TCA angle. This dataset also includes TCA angles that have been pre-computed on each image for model training. After establishing the model, we assessed its performance on a separate dataset from CHOP. In order to validate the performance of our method on a broad range of local images, our study included 83 patients who underwent multilevel posterior spinal fusion (PSF) for AIS correction. Demographics and baseline characteristics are included in Table 1.

**Table 1:** Baseline and surgical characteristics of the validation cohort undergoing long-segment posterior spinal fusion for adolescent idiopathic scoliosis correction

Variables	Overall (n = 83)
Age	13 (11–14)
Female sex	61 (73.5%)
Race	
White	56 (67.5%)
Black	18 (21.7%)
Other	9 (10.8%)
Hispanic ethnicity	6 (7.2%)
BMI (kg/m2)	21.6 (19.4–25.8)
Prior non-surgical intervention	51 (61.4)
Radiographic measurements	
Right TC	82 (98.8)
TCA (°)	56.0 (52.0–64.0)
Left LC	82 (98.8)
LCA (°)	42.0 (32.0–52.0)
PSF (levels)	11 (10–12)
PSO (levels)	0 (0–4)

Data are median (interquartile range) or number of patients (%)

*BMI* Body-mass-index, *TC* Thoracic curve, *TCA* Thoracic cobb angle, *LC* Lumbar curve, *LCA* Lumbar cobb angle. *PSF* Posterior spinal fusion, *PSO* Pedicle subtraction osteotomy

**Fig. 2:**
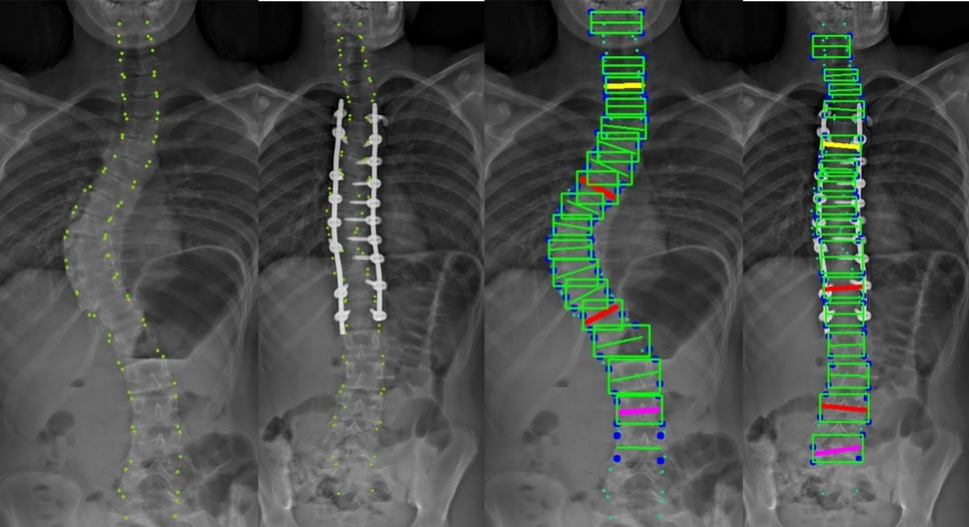
Whole-spine standing radiographs of a patient undergoing long-segment posterior spinal fusion for adolescent idiopathic scoliosis correction. The images display preoperative coronal annotations (**A**), postoperative coronal annotations (**B**), preoperative coronal predictions (**C**), and postoperative coronal predictions (**D**), including keypoints and bounding boxes

**Fig. 3:**
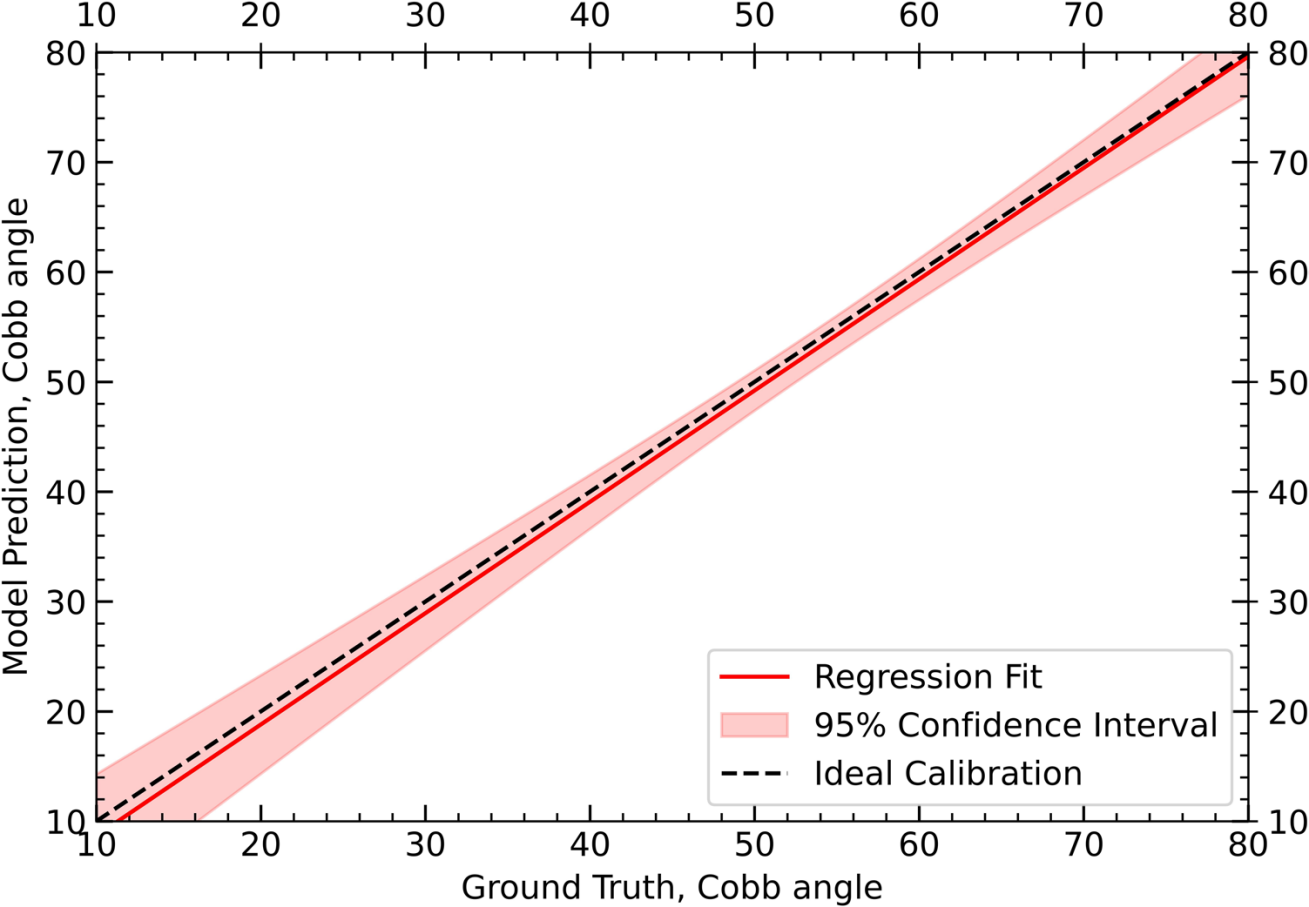
Regression calibration plot of the validation cohort with 95% confidence interval

Radiographic images from our institutional registry used for validation were in the standard Digital Imaging and Communications in Medicine (DICOM) format. Coronal whole-spine standing radiographs had a mean resolution of 4264 × 1980 with a mean dots per inch (dpi) value of 135. Representative pre-operative and post-operative coronal images from one patient, including annotations and predictions are shown (Fig. 2). We assessed the performance of the automated method by comparing the KR-CNN results to values calculated by board-certified radiologists. Our model had a MAE of 2.22 (95% confidence interval: 1.06—3.39), MedAE of 1.47 (0.89–3.15), MSE of 9.1, ICC of 0.98, and SMAPE of 4.29 (Table 2). A regression calibration plot is illustrated (Fig. 3).

**Table 2:** Performance metrics of the keypoint region-based convolutional neural network (KR-CNN) including external and internal validation for automated measurement of thoracic Cobb angles in the validation cohort of patients undergoing long-segment posterior spinal fusion for adolescent idiopathic scoliosis correction

Model	MAE ± SD (95% CI)	MedAE (IQR; 95% CI)	MSE	SMAPE	ICC GT
KR-CNN Resnet 50 (Current Study, External Validation)	2.22 ± 2.04 (1.06 to 3.39)	1.47 (0.73–2.97; 0.89 to 3.15)	9.1	4.29	0.98
KR-CNN Resnet 50 (Current Study, Internal Validation)	1.78 ± 1.61 (1.12 to 2.44)	1.21 (0.65–2.43; 0.81 to 2.74)	6.84	3.67	0.99
Landmark Net	13.85	—	—	38.87	—
Angle Net	9.36	—	—	23.91	—
AEC-Net	4.90	—	—	23.59	—
Vertebra Landmark Detection	—	—	—	10.81	—
Auto-CA	—	—	—	5.27	—
VLTENet	2.51	—	—	5.44	—
RegNet	2.92	—	—	6.87	
MVE-Net	7.81			24.94	
Seg4Reg	—	—	—	21.71	—
ScolioVis	—	—	—	8.97	—
Minimum bounding rectangle	—	—	—	—	0.97

*ICC* Intraclass correlation coefficient, *GT* ground truth, *IQR* interquartile range, *MAE* Mean absolute error, *MedAE* Median absolute error, *MSE* Mean squared error, *KR-CNN* Keypoint Region-based convolutional neural network, *Resnet* 50 residual network 50, *SD* Standard deviation, *SMAPE* Symmetric mean absolute percentage error

### Secondary outcomes

These results demonstrated a notable improvement in accuracy compared to existing automated TCA measurement methods, including AEC-Net [3], Seg4Reg [19], ScolioVis [30], Auto-CA [28], VLTNet [42] and others [1, 3, 12, 18, 33, 41]. These previous methods utilized a variety of deep learning frameworks including direct Cobb angle regression and methods based on landmark detection and vertebra segmentation. The SMAPE is a commonly reported performance metric for automated measurement methods, and our value of 4.29 is lower than existing methods, outperforming Auto-CA which had the second lowest SMAPE value of 5.27. Similarly, the MAE of 2.22 achieved in this study outperformed the methods that reported this metric, including VLTENet, which achieved the second lowest value of 2.51. Moreover, by demonstrating the performance on external data from the training set, our method uniquely demonstrated that this superior performance metrics are generalizable across institutions, while most methods report validation metrics by splitting a single dataset into training and testing subset. For example, VLTENet, which achieved a SMAPE of 5.44 on a test subset of the AASCE dataset, this value dropped to 13.9 when the method was applied to an external clinical dataset [42]. Taken together, we report a method using a KR-CNN for automated TCA measurement that outperformed existing methods and demonstrated robust accuracy on external data.

## Discussion

In this study we developed an KR-CNN for the automated measurement of the thoracic Cobb angle. The benchmarking results of our method indicated that the implementation of this machine learning algorithm yielded precise TCA measurements, demonstrating its feasibility to assist in the screening, evaluation, and early intervention in AIS cases. Previous research has demonstrated the application of convolutional neural networks (CNNs) for automating these measurements with high accuracy and consistency [11, 22]. These studies highlight the potential of AI to overcome the limitations of manual measurement, characterized by high interobserver variability and potential for human error. Recently, there have been advances in R-CNN model implementations that had promising results [20, 21, 30]. However, these methods have not been widely adopted into clinical practice because of their lower accuracy and lack of generalizability. Our study presents a model that provides superior performance and enhanced generalizability. Our framework is adaptable and can be readily trained on a broad range of datasets to optimize the results at the level of specific institutions or even individual users.

The model demonstrated exceptional performance across all metrics, particularly in MAE and SMAPE, which outperformed outcomes reported in recent literature. The high ICC further establishes its reliability. These findings suggest that the model's accuracy and consistency are noteworthy within the current landscape of automated spinal measurements. Before clinical integration, thorough validation is essential, emphasizing the model’s potential to enhance the reliability and efficiency of clinical decision-making processes.

Convolutional neural network approaches hold broad potential for their clinical applicability. Previous studies have demonstrated the utility of R-CNN methods for fast and accurate radiographic measurement and feature detection in a range of contexts, ranging from periodontics [2, 32] to neurosurgery [24, 38] to pathology [29]. In these disparate contexts, AI-assisted image analysis may improve both efficiency and accuracy in tasks involving the identification of structures and feature measurements. Factors such as human error, variability in anatomic landmarks, and differences in imaging quality can yield a striking degree of error in conceptually straightforward tasks. AI technologies can detect small changes in background intensity at the level of individual pixels, rendering these approaches far more sensitive to identify patterns with high confidence that are too subtle or microscopic for a human observer to discern. A strength of CNNs is their ability to define object boundaries and edges at the pixel level. This enables highly consistent measurements of distances and quantification of shape features on radiographic images, abrogating potential concerns for inter-rater reliability. Collectively, improving diagnostic accuracy has far-reaching consequences, including improvements in clinical decision-making, surgical planning, and post-operative follow-up.

Overall, recent advancements in deep learning-based landmark detection have focused on improving accuracy, reliability, and adaptability [16, 17, 40]. New models, such as multi-task learning networks, simultaneously detect key landmarks and segment anatomical structures, reducing the need for multiple separate models [40]. Others, like FDGR-Net, refine landmark detection by filtering out irrelevant background noise and improving focus on subtle anatomical features [17]. Although vision learning models, a type of multimodal deep learning model, may serve a crucial role in medical imaging, there are several deficiencies in clinical applicability, costs, and data sharing and.protection [15]. In spinal imaging, landmark-aware networks enhance Cobb angle measurement by improving feature detection and minimizing the impact of imaging artifacts. These innovations, along with our findings, highlight the growing potential of CNN models to automate medical imaging tasks and integrate seamlessly into clinical workflows.

### Limitations

The findings of the present methodological feasibility study should be interpreted in the context of its limitations. Although the model was rigorously trained and validated, its population—AIS patients—may be considered skewed relative to the general population. However, since AIS imaging often presents greater interpretative challenges, particularly due to included hardware, the model is expected to generalize well, performing at least as accurately, if not better, in broader cohorts. Nonetheless, further external validation is necessary to confirm its robustness and clinical applicability. While our imaging dataset is comprehensive, including a broad range of coronal TCA measurements, the inclusion of more images across various populations and retraining the model using United States data, followed by external validation, could improve generalizability. Fine-tuning the KR-CNN with data augmentations could further enhance model robustness. Nonetheless, by using our institutional data, including a wide range of preoperative and postoperative X-ray images, as a testing dataset, we already demonstrated external validity beyond the larger registry of public data that was used to train the model. We acknowledge the critical importance of data governance and recognize that future implementation studies must focus on curating governable datasets that ensure appropriate access and oversight. Additionally, other spinal parameters have been associated with AIS outcomes that could be included in the model, including but not limited to location of apical vertebra/disc, convexity of the curve, thoracic kyphosis, lumbar lordosis, and pelvic tilt [39]. Another limitation is the potential for the model to encounter difficulties with outliers or atypical cases, which may not be adequately represented in the training data. The automated approach, while highly accurate in standard cases, could struggle with images that present unique challenges, such as severe deformities, poor image quality, or unusual anatomical variations. This underscores the need for further studies to assess the model’s performance and suggests that the implementation of a confidence output that could estimate the level of error in the given prediction and flag outliers for manual review would be a beneficial addition in future iterations. Moreover, the utilization of multiple imaging views, in addition to the standard coronal view, could further improve accuracy, especially in outlier cases. This could ultimately be extended to incorporate three-dimensional imaging and longitudinal data. Finally, widespread adoption into clinical practice remains a challenge for any AI model, including the KR-CNN. Factors such as integration with existing clinical workflows, user acceptance, and the need for continuous monitoring and updates of the model as new data becomes available must be carefully considered before widespread clinical application.

## Conclusion

Overall, this study highlights the precision and reliability of AI-based automation of spinal imaging interpretation through the development of a KR-CNN for thoracic Cobb angle measurement, while providing a framework for further optimization and application of this dynamic model. Future research should focus on expanding datasets, refining the algorithm, conducting rigorous external validations, clinical impact, and integration. Ultimately, the successful implementation of an automated workflow for spinopelvic measurement has the potential to reduce costs, limit human error, and improve patient outcomes.

## Data Availability

In accordance with the University of Pennsylvania Institutional Review Board requirements, the research data supporting this project may not be publicly shared. De-identified data may be shared upon reasonable request following execution of a Data Usage Agreement. Please reach out to corresponding author, M.M.D., with any inquiries.
